# Electrophysiological, structural, and functional disorders in patients with inflammatory cardiomyopathy secondary to inflammatory myopathy

**DOI:** 10.1111/anec.12938

**Published:** 2022-02-20

**Authors:** Yingxian Liu, Jeffrey Hsu, Xiaohang Liu, Xue Lin, Yanlin Zhu, Fuwei Jia, Wei Wu, Wei Chen, Qian Wang, Ligang Fang

**Affiliations:** ^1^ Department of Cardiology State Key Laboratory of Complex Severe and Rare Diseases Peking Union Medical College Hospital Chinese Academy of Medical Sciences and Peking Union Medical College Beijing China; ^2^ Department of Rheumatology State Key Laboratory of Complex Severe and Rare Diseases Peking Union Medical College Hospital Chinese Academy of Medical Sciences and Peking Union Medical College Beijing China

**Keywords:** electrocardiogram, idiopathic inflammatory myopathies, inflammatory cardiomyopathy, prognosis

## Abstract

**Background:**

Inflammatory cardiomyopathy (IC) is a syndrome with chronic myocarditis and cardiac remodeling. This study aimed to explore predicting factors of adverse outcomes in patients with IC secondary to idiopathic inflammatory myopathy (IIM‐IC).

**Methods:**

By means of a single‐center retrospective study, 52 patients with IIM‐IC at Peking Union Medical College Hospital were identified from January 1999 to June 2019. Electrocardiogram and echocardiography data were analyzed for the primary outcome (defined as all‐cause death) and secondary outcomes (defined as re‐hospitalization of heart failure and all‐cause death), using regression and survival analysis.

**Results:**

The prevalence of atrial fibrillation, ventricular tachycardia, Q‐wave abnormality, left ventricular conduction abnormalities, and reduced left ventricular ejection fraction (LVEF) (≤40%) were 65.4%, 67.3%, 67.3%, 61.6%, and 50.5%. After a median follow‐up of 2 years (IQR 0.8–3.0), 26 cases were readmitted due to heart failure. Twenty‐two deaths were recorded, including 20 cardiogenic deaths. Among the patients with adverse events, the incidence of poor R‐wave progression, low‐voltage of the limb leads, Q‐wave abnormality, QRS duration >130 ms, left ventricular enlargement, and impaired systolic function were higher. Kaplan–Meier analysis showed that Q‐wave abnormality, limb leads low‐voltage, LVEF ≤40%, and left ventricular end‐diastolic dimension >60 mm were correlated with shorter survival. However, multivariate Cox regression analysis revealed that only Q‐wave abnormality (*HR* = 12.315) and LVEF ≤40% (*HR* = 5.616) were independent risk factors for all‐cause death.

**Conclusion:**

Q‐wave abnormality and reduced LVEF are predictive of poor prognosis in patients with IIM‐IC.

## Graphical abstract

Systemic evaluation of IC in patients with IIM.

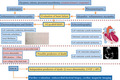

## 1 INTRODUCTION

Inflammatory cardiomyopathy (IC) is a clinical syndrome defined as myocarditis concomitant with systolic and/or diastolic dysfunction and ventricular remodeling (Caforio et al. 2013). Myocarditis may be diagnosed clinically, by imaging and abnormal cardiac biomarkers, or pathologically, by endomyocardial biopsy showing inflammatory infiltration and nonischemic myocyte injury (Aretz Ht et al, 1987). IC can be due to a variety of etiologies, including infectious, autoimmune, or idiopathic (Caforio et al. 2013). Autoimmune myocarditis is reported increasingly in patients with rheumatic diseases such as systemic lupus erythematosus, systemic sclerosis, and idiopathic inflammatory myopathies (IIM). Among them, IIM is a series of rare immune‐mediated inflammatory diseases mainly including dermatomyositis and polymyositis (Hoogendijk et al., 2004). In IIM, skeletal muscular injury is typical while myocarditis can also be concomitant. The frequency of electrocardiogram changes could be observed in 72% of IIM patients (Lundberg 2006), and autopsy show that about one‐third of cases with IIM have subclinical myocardial changes (Denbow et al. 1979). Moreover, cardiac injury was cited as the primary cause of death in 10‐20% of IIM patients (Gupta et al. 2011). When heart failure (HF) or fatal arrhythmia complications happened, the mortality could reach 46.3% in IIM (Zhang et al. 2012), and the cardiac dysfunction was a predictive factor of poor prognosis in IIM (Marie 2012).

It is valuable to identify electrophysiological abnormalities and subclinical heart impairment in IIM (Mahrholdt et al. 2004) and to recognize patients with IC from patients with primary DCM. Previous studies reported that the most common cardiac features of polymyositis and dermatomyositis were arrhythmia and diastolic HF (Guerra et al. 2017), while cardiomyopathy and systolic HF were rare (Gupta et al. 2011). In fact, reports of prognostic relevance of electrocardiogram in patients with IIM‐associated IC (IIM‐IC) are scant.

Here, we presented a retrospective cohort study of IIM‐IC patients, analyzed their electrocardiographic and echocardiographic characteristics, and followed up with them to explore the possible predicting factors of adverse outcomes.

## 2 METHODS

### 2.1 Patient selection

Fifty‐two consecutive patients diagnosed with IC and IIM at our hospital between 1999 and 2019 were retrospectively studied. All the cases were transferred from other hospitals, because of the complexity and difficulty of their diseases. The diagnosis of IC was based on the presence of myocarditis and DCM. Myocarditis was confirmed by typical clinical presentations, newly abnormal electrocardiographic features, elevated cardiac troponins, functional, and structural abnormalities on cardiac imaging, according to the 2013 ESC position statement of myocarditis (Caforio et al. 2013). Definition of DCM was in accordance with the 2007 ESC classification of cardiomyopathies, characterized by left ventricular dilation, and impaired systolic function (Elliott et al. 2008). Serum level of N‐terminal brain natriuretic peptide precursor (NT‐proBNP) was higher than 400 ng/L in all involved patients (Yancy et al. 2013). According to medical history, echocardiography, and coronary angiography, patients with familial histories of cardiomyopathy, or other organic heart diseases such as coronary heart disease, congenital disease, or rheumatic heart disease were excluded. Patients who were lost to follow‐up and who were concomitant with malignant tumors or chronic kidney disease (defined as estimated glomerular filtration rate < 45 ml/min) were also excluded.

**FIGURE 1:**
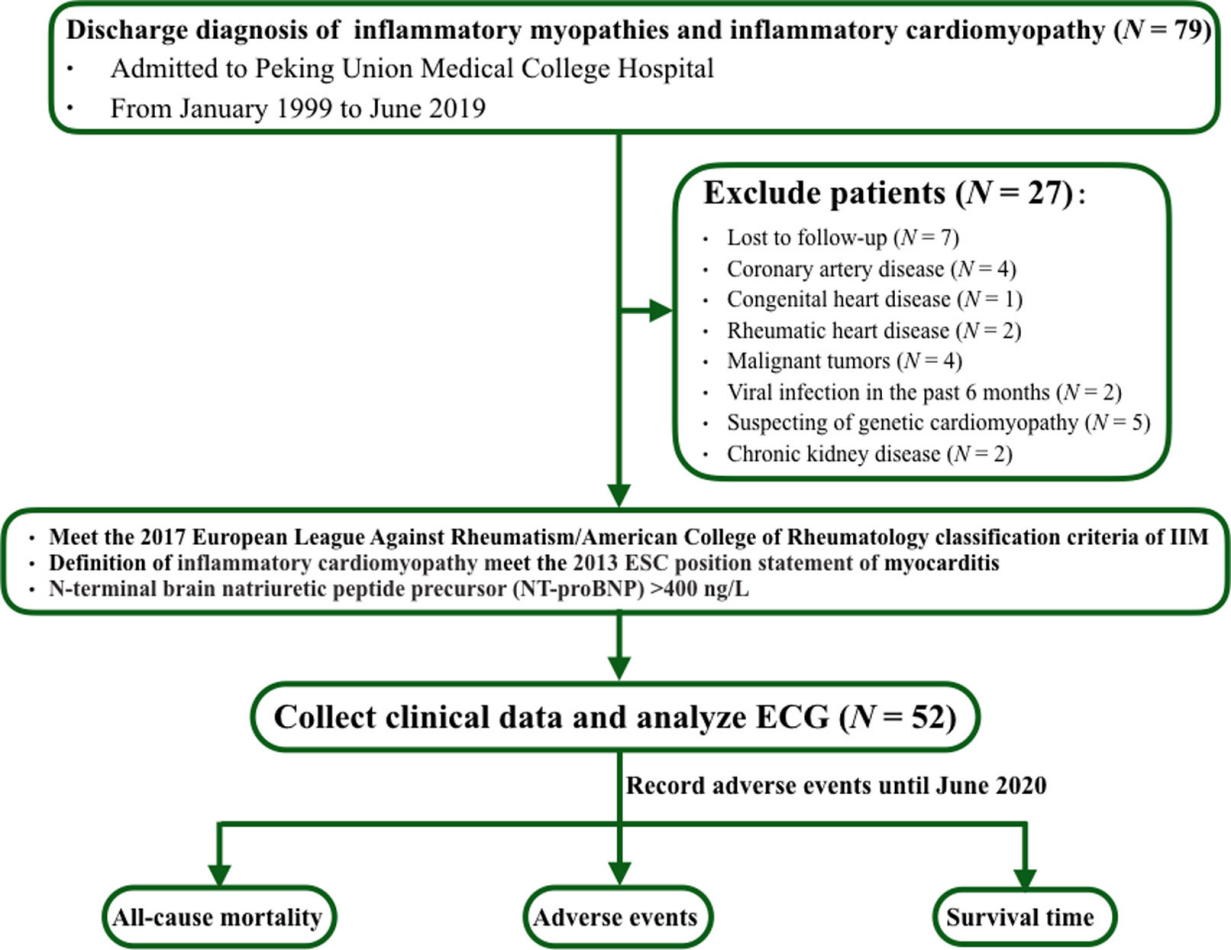
Flow diagram of the patients included in the study

The diagnosis of polymyositis and dermatomyositis was based on the 2017 European League Against Rheumatism/American College of Rheumatology classification criteria for IIM, with a definite diagnosis of IIM according to muscle biopsies, when the total aggregate score was 8.7 or higher corresponding to a probability of at least 90% (Bazzani et al., 2010). Muscle biopsies were assessed in the neuropathological laboratory of PUMCH with accepted histopathological diagnostic criteria (Bazzani et al., 2010). The diagnosis of IIM‐IC was confirmed through multidisciplinary consultations of cardiologists, rheumatologists, radiologists, and pathologists. Figure S1 represents a typical example of electrocardiogram, echocardiography, cardiovascular magnetic resonance (CMR), and result of quadriceps muscular biopsy. The study flow chart is shown in Figure 1. This study is a part of the study “Diagnostic and imaging indicators of immunocardiomyopathic patients,” which has been registered in Clinical trials (NCT 03885375), and is conducted with the approval of the Institutional Review Board of Peking Union Medical College Hospital. Due to the retrospective nature of the study, informed consent is waived.

### 2.2 Data collection

The baseline clinical data, including demographic data (such as disease onset age and gender), discharge diagnoses, disease duration, clinical manifestations, NYHA cardiac functional classification, and treatment strategies, were recorded. Initial electrocardiographic images and echocardiographic reports during their first hospitalization were analyzed by 2 cardiologists who were blinded of the patient’s illness.

### 2.3 Electrocardiographic and echocardiographic data

The baseline electrocardiographic variables were analyzed: resting heart rate, electronic axis and voltage, QRS duration (normal <120 ms), corrected QT duration (normal range 330–440 ms), and presence and distribution of Q‐wave abnormality. Other arrhythmias such as conduction disturbance, atrial fibrillation (AF), and ventricular tachycardia were also recorded. According to echocardiography (GE Medical Systems,), left ventricular end‐diastolic dimension (LVEDD), left ventricular fractional shortening, and left ventricular ejection fraction (LVEF, modified Simpson method), tricuspid regurgitation peak velocity (TRV), ventricular wall motions, and presence or absence of pericardial effusion were recorded. Reduced LVEF was defined as LVEF less or equal to 40% (Ponikowski et al., 2016). Impaired left ventricular diastolic function was defined as more than half of following abnormalities: decreased annular e’ velocity, elevated average E/é, left atrial enlargement, and increased TRV (>2.8 m/s) (Nagueh et al., 2016).

### 2.4 Follow‐up and endpoints

Structured surveys were conducted through medical record reviews and outpatient visits, and data were supplemented by family report via phone to capture deaths outside the hospital. All cases were followed up until June 2020. Primary endpoint was defined as all‐cause death, and secondary endpoints were defined as adverse events including all‐cause death and HF requiring re‐admission. The overall survival time was defined as the duration from initial symptoms onset to death.

### 2.5 Statistical analysis

Data were analyzed using the SPSS version 19.0 software (SPSS Inc.,). All data were expressed as mean ± standard deviation (SD) and median (interquartile range, IQR) for normally and non‐normally distributed data, respectively, and as a percentage when appropriate. To compare difference between groups, independent samples T‐tests and Mann–Whitney tests were used for continuous variables that were normal and not normally distributed, respectively. The chi‐squared or Fisher’s exact tests were used for categorical data as appropriate. Univariate and multivariate binary logistic regression model was used to identify factors associated with adverse events. The possible risk factors were evaluated by Kaplan–Meier survival analysis along with log‐rank tests. *p* value <.05 (two‐tailed) was considered statistically significant.

## 3 RESULTS

### 3.1 Study population

The clinical characteristics and treatment strategies of the study patients are shown in Table 1. Of the 52 patients included, there were 19 men and 33 women. The mean age was 48.4 ± 1.8 years (range from 16 to 69 years). There were 34 cases with polymyositis and 18 cases with dermatomyositis. The chief complaint reported were dyspnea, palpitation, and skeletal muscular weakness.

**TABLE 1:** Clinical data of patients with idiopathic inflammatory myopathy and inflammatory cardiomyopathy

Clinical parameters	Total (*N* = 52)	No of events* (*N* = 23)	Adverse outcome^&^ (*N* = 29)	*p* value
Female	33 (63.5%)	14 (60.9%)	19 (65.5%)	.730
Age of onset, years	48.4 ± 1.8	49.3 ± 11.5	47.7 ± 14.5	.161
Disease course, years	2.0 (0.9–4.8)	2.5 (0.9–5.3)	2.0 (1.0–4.8)	.963
Hypertension	14 (26.9%)	7 (30.4%)	7 (24.1%)	.611
Hyperlipidemia	5 (9.6%)	1 (4.3%)	4 (13.8%)	.368
Diabetic mellitus	5 (9.6%)	1 (4.3%)	4 (13.8%)	.368
Smoking	9 (17.3%)	4 (17.4%)	5 (17.2%)	1.000
Obesity	1 (1.9%)	1 (4.3%)	0	.442
Palpitation	42 (80.8%)	19 (82.6%)	23 (79.3%)	1.000
Dyspnea	46 (88.5%)	19 (82.6%)	27 (93.1%)	.387
Syncope	10 (19.2%)	4 (17.4%)	6 (20.7%)	1.000
NYHA class III‐IV	36 (69.2%)	13 (56.5%)	23 (79.3%)	.006
Polymyositis	34 (65.4%)	17 (73.9%)	17 (58.6%)	.250
Proximal muscular weakness	41 (78.8%)	20 (87.0%)	21 (72.4%)	.308
Rash	19 (36.5%)	8 (34.8%)	11 (37.9%)	.815
Interstitial lung disease	20 (38.5%)	10 (43.5%)	10 (34.5%)	.574
Pulmonary hypertension	19 (36.5%)	6 (26.1%)	13 (44.8%)	.199
Creatine kinase, U/L	883.0 (344.8–1953.0)	472.0 (166.5–1789.8)	883.0 (653.3–2840.3)	.143
Creatine kinase isozyme, µg/L	23.6 (7.0–57.8)	14.5 (3.1–47.4)	24.1 (9.7–52.3)	.233
Cardiac troponin I, µg/L	0.3 (0.1–1.9)	0.4 (0.2–2.7)	0.2 (0.1–3.9)	.705
Lactate dehydrogenase, U/L	396.0 (335.8–572.8)	420.0 (332.0–512.5)	396.0 (349.0–666.8)	.919
Hs‐CRP, mg/L	6.4 (1.5–23.0)	7.2 (1.8–27.2)	9.0 (2.4–16.1)	.545
NT‐proBNP, ng/L	3689.0 (1605.5–7339.0)	1940.5 (953.5–4146.3)	4690.5 (2450.0–9257.0)	.008
AMA‐M2	12 (23.1%)	5 (21.7%)	7 (24.1%)	1.000
Anti‐cardiolipin antibody	4 (7.7%)	3 (13.0%)	1 (3.4%)	.310
Jo‐1 antibody	3 (5.8%)	1 (4.3%)	2 (6.9%)	1.000
Ro‐52 antibody	18 (34.6%)	10 (43.5%)	8 (27.6%)	.232
Glucocorticoid shock therapy	17 (32.7%)	9 (39.1%)	8 (27.6%)	.426
Methotrexate	23 (55.2%)	8 (34.8%)	15 (51.7%)	.222
Cyclophosphamide	23 (44.2%)	11 (47.8%)	12 (41.4%)	.642
Cyclosporine	12 (23.1%)	6 (26.1%)	6 (20.7%)	.646
ACEI/ARB	31 (59.6%)	11 (47.8%)	20 (69.0%)	.123
Beta blockers	41 (78.8%)	19 (82.6%)	22 (75.9%)	.735
Spironolactone	32 (61.5%)	13 (56.5%)	19 (65.5%)	.508

Note

For continuous variables: Independent sample T test or Mann–Whitney tests. For categorical data: chi‐squared or Fisher’s exact test.

*Defined as patients survived without any re‐hospitalization of HF. & Defined as patents with re‐hospitalization of HF or all‐cause death.

Abbreviations: Hs‐CRP, High‐sensitivity C‐reactive protein; NT‐proBNP, N‐terminal pro‐brain natriuretic peptide; ACEI, Angiotensin‐converting enzyme inhibitors; ARB, Angiotensin receptor blockers.

All the involved patients were followed for a median of 2 years (IQR 0.8–3.0 years) and 22 deaths were recorded. Cardiogenic death was noticed in 20 cases; the other two cases of deaths were caused by infective complication and progressive respiratory failure, respectively. Twenty‐six cases were readmitted due to HF and five cases were implanted with implanted cardiac device, including 3 of implantable cardioverter defibrillator. IIM‐IC patients that required rehospitalization for HF or suffered from all‐cause death had worse baseline functional status (greater percentage NYHA Class III‐IV) and higher levels of NT‐proBNP compared to IIM‐IC patients who did not have adverse events (Table 1).

### 3.2 Electrophysiological features

#### 3.2.1 Rhythm and rate

**FIGURE 2:**
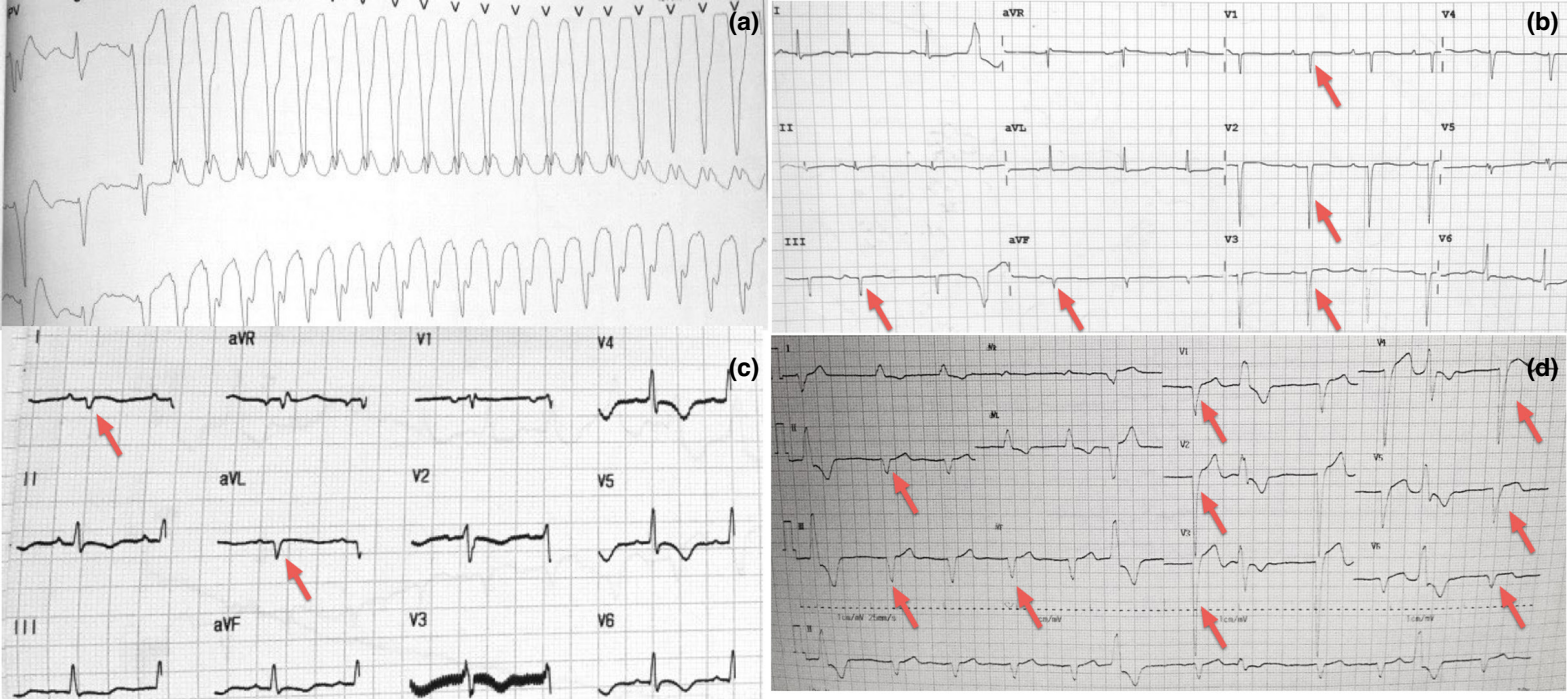
Electrocardiogram characteristics of patients with IIM‐IC. (a) A 54‐year‐old female with polymyositis. A sustained ventricular tachycardia persisted for 18 min and 10 s, with a ventricular rate of 214 bpm. (b) A 38‐year‐old female with polymyositis, electrocardiogram showed premature ventricular compaction, left deviation of electronic axis, low voltage of the limb leads, and Q‐wave abnormality (red arrow). (c) A 25‐year‐old female with polymyositis and died of cardiogenic shock. Electrocardiogram showed prolonged corrected QT interval (470 ms), low voltage of the limb leads, abnormal Q waves (red arrow), and diffuse T‐wave inversion. (d) A 67‐year‐old female with polymyositis. Electrocardiogram showed persistent AF, premature ventricular compaction, left anterior fascicular block, abnormal Q waves (red arrow), and prolonged QRS duration (149 ms)

The average heart rate recorded by the electrocardiogram was 82 ± 19 beats per minute. Pause greater than 2 seconds in sinus rhythm or greater than 3 seconds in AF and advanced atrioventricular block were noticed in 11 and 2 patients, respectively. Premature ventricular contraction was present in 90.4% of involved patients, while ventricular tachycardia could be seen in more than two thirds of our cases (67.3%) (Figure 2a,b,d). Most of the patients manifested atrial arrhythmia, including paroxysmal AF (40.4%), persistent AF (25.0%), and atrial flutter (11.5%) (Figure 2d). No difference of above parameters could be observed between patients with or without adverse events (Table 2).

**TABLE 2:** Electrocardiographic and echocardiographic characteristics of patients with or without adverse outcome

Parameter	Total (*N* = 52)	No events (*N* = 23)	Adverse outcome (*N* = 29)	*p* value
Average heart rate on Holter, bpm	82.1 ± 19.4	82.1 ± 17.3	82.1 ± 21.3	.993
Cardiac arrest	11 (21.2%)	5 (21.3%)	6 (20.7%)	.927
QRS duration >130 ms	18 (34.6%)	4 (17.4%)	14 (48.3%)	.038
Corrected QT interval >440 ms (%)	32 (61.5%)	13 (56.5%)	19 (65.5%)	.508
Electronic axis and voltage				
Left axis deviation	36 (69.2%)	14 (60.9%)	22 (75.9%)	.245
Right axis deviation	8 (15.4%)	2 (8.7%)	6 (20.7%)	.234
High‐voltage of the precordial leads	11 (21.2%)	5 (21.7%)	6 (20.7%)	.927
Poor R‐wave progression on precordial leads	35 (67.3%)	12 (52.2%)	23 (79.3%)	.038
Low‐voltage of the limb leads	25 (48.1%)	7 (30.4%)	18 (62.1%)	.023
Conduction abnormalities				
Left anterior fascicular block	14 (26.9%)	8 (34.8%)	6 (20.7%)	.255
Left bundle branch block	7 (13.5%)	2 (8.7%)	5 (17.2%)	.370
Right bundle branch block	6 (11.5%)	3 (13.0%)	3 (10.3%)	1.000
Intraventricular block	11 (21.2%)	3 (13.0%)	8 (27.6%)	.308
Multibundle branch block	6 (11.5%)	2 (8.7%)	4 (13.8%)	.682
Advanced atrioventricular block	2 (3.8%)	1 (4.3%)	1 (3.4%)	1.000
Atrial arrhythmia				
Atrial tachycardia	13 (25.0%)	6 (26.1%)	7 (24.1%)	.872
Paroxysmal atrial fibrillation	21 (40.4%)	9 (39.1%)	12 (41.4%)	.870
Persistent atrial fibrillation	13 (25.0%)	5 (21.7%)	8 (27.6%)	.629
Atrial flutter	6 (11.5%)	4 (17.4%)	2 (6.9%)	.387
Q‐wave abnormality	35 (67.3%)	12 (52.2%)	23 (79.3%)	.038
Anterior wall (V3‐V6)	13 (25.0%)	3 (13.0%)	10 (34.5%)	.110
Anterior–septal wall (V1‐V3)	21 (40.4%)	9 (39.1%)	12 (41.4%)	.870
Inferior wall (II, III, AVF)	13 (25.0%)	2 (8.7%)	11 (37.9%)	.023
Lateral wall (I, AVL, V5)	3 (5.8%)	1 (4.3%)	2 (6.9%)	1.000
Ventricular arrhythmia				
Premature ventricular compaction (%)	47 (90.4%)	20 (87.0%)	27 (93.1%)	.644
Nonsustained ventricular tachycardia (%)	29 (55.8%)	11 (47.8%)	18 (62.1%)	.304
Sustained ventricular tachycardia (%)	6 (11.5%)	2 (8.7%)	4 (13.8%)	.682
Echocardiography				
Left ventricular end‐diastolic dimension, mm	53.7 ± 7.9	51.4 ± 5.9	55.3 ± 8.9	.078
Left ventricular ejection fraction (Biplane), %	37.8 ± 11.5	44.0 ± 10.6	33.1 ± 9.7	<.001
Reduced left ventricular ejection fraction	26 (50.0%)	6 (26.1%)	20 (69.0%)	.002
Impaired left ventricular diastolic function	29 (55.8%)	11 (47.8%)	18 (62.1%)	.304
Pulmonary arterial systolic pressure, m/s	38.3 ± 13.0	34.6 ± 11.3	41.3 ± 13.4	.060
Pericardial effusion	27 (51.9%)	11 (47.8%)	16 (55.2%)	.780

Reduced left ventricular ejection fraction, defined as left ventricular ejection fraction ≤40%. Impaired left ventricular diastolic function, defined as more than half of following abnormalities: decreased annular e’ velocity (septal e’<7 cm/s, lateral e’<10 cm/s), elevated average E/é (>14), left atrial enlargement (volume index> 34 ml/m^2^), and increased tricuspid regurgitation velocity (>2.8 m/s).

#### 3.2.2 Waveform

More than two thirds of the patients showed left deviation of electrical axis (69.2%) at baseline (Figure 2b,d), the prevalence of Q‐wave abnormality was 67.3% (Figure 2b,c,d), and the cumulative probability of left ventricular conduction abnormalities, which included left anterior fascicular block ((Figure 2d), left bundle branch block, and intraventricular block, was 61.6%. Prolonged corrected QT interval >440 ms was found in 61.5% of these patients. When compared to patients without adverse events, patients with adverse events were more likely to have poor R‐wave progression on precordial leads (52.2% vs. 79.3%, *p* = 0.038) (Figure 2b,2d), low‐voltage of the limb leads (30.4% vs. 62.1%, *p* = 0.023) (Figure 2b,c), Q‐wave abnormality (52.2% vs. 79.3%, *P* = 0.038) (Figure 2b,c,d) and QRS duration >130 ms (17.4% vs. 48.3%, *p* = 0.038) (Figure 2d) at baseline (Table 2).

#### 3.2.3 Cardiac structural and functional features

The echocardiographic reports of all patients were consistent with the morphological feature of DCM and were mainly manifested as mildly dilated left ventricle (LVEDD: 53.7 mm ± 7.9 mm) and impaired left ventricular systolic function (LVEF: 37.8% ± 11.5%). Twenty‐six patients (50.5%) met the definition of reduced LVEF. Left ventricular diastolic dysfunction was common (29/52). There were 30 patients with left ventricular enlargement, with a LVEDD of 53.7 ± 7.9 mm (Table 2). A typical example of echocardiography is shown in Figure S1C.

#### 3.2.4 Association between cardiac parameters and adverse events

Univariate Cox regression analyses were used to explore the electrocardiographic parameters predictive of adverse events. Q‐wave abnormality (*HR* = 3.545, *95%CI* 1.027–12.236), low voltage of the limb leads (*HR* = 2.804, *95%CI* 1.137–6.916), LVEDD >60 mm (*HR* = 2.462, *95%CI* 1.014–5.978), and reduced LVEF (*HR* = 3.247, *95%CI* 1.076–9.795) were significantly associated with adverse events (*p* < .05, respectively). However, after adjusted for baseline NYHA cardiac function and serum levels of NT‐proBNP in multivariate Cox regression model, only Q‐wave abnormality (*HR* = 12.315, *95%CI* 1.191–127.374, *p* = .035) and reduced LVEF (*HR* = 5.616, *95%CI* 1.256–25.109, *p* = .024) were the independent predictors for adverse events (Table 3).

**TABLE 3:** Cox regression analyses between all‐cause death and parameters of electrocardiogram and echocardiography (*N* = 52)

Parameter	Univariate			Multivariate adjusted*		
	Exp (B)	95%CI	*p* value	Exp (B)	95%CI	*p* value
Q‐wave abnormality	3.545	1.027–12.236	0.045	12.315	*1.191*–*127.374*	.*035*
Poor R‐wave progression on precordial leads	1.588	0.577–4.369	0.370			
Low voltage of the limb leads	2.804	1.137–6.916	0.025			
QRS duration >130 ms	1.003	0.419–2.402	0.994			
Left ventricular end‐diastolic dimension >60 mm	2.462	1.014–5.978	0.046			
Left ventricular ejection fraction ≤40%	3.247	1.076–9.795	0.037	5.616	1.256–25.109	.024
Pulmonary arterial systolic pressure	1.019	0.987–1.053	0.248			

*Adjusting for baseline NYHA cardiac function and NT‐proBNP.

**FIGURE 3:**
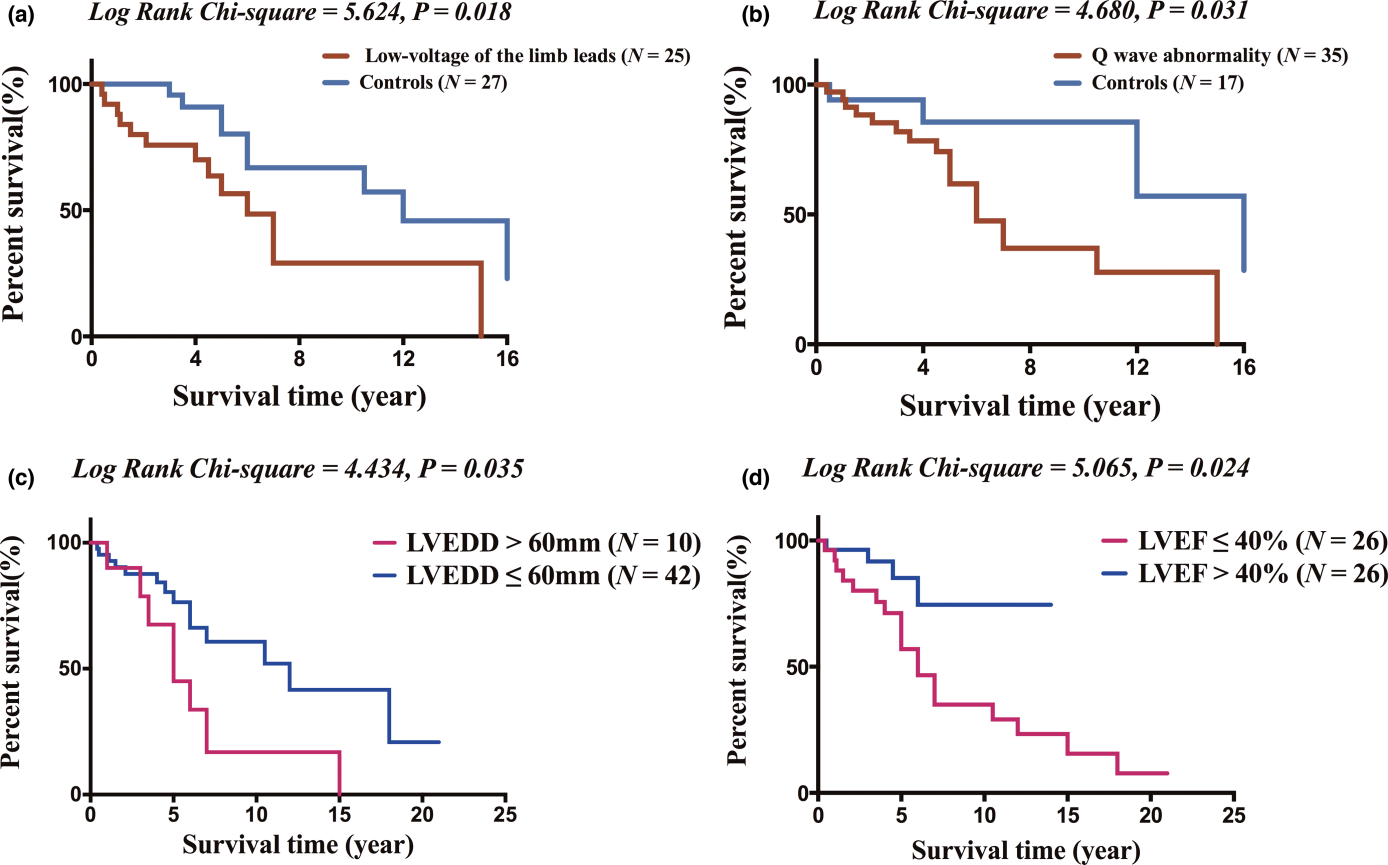
Kaplan–Meier Survival curve of all‐cause mortality in patients with IIM‐IC. Comparisons between (a), patients with low voltage of the limb leads and controls without low voltage of the limb leads; (b), patients with Q‐wave abnormality and controls without Q‐wave abnormality; (c), patients with LVEDD >60 mm and LVEDD ≤60 mm; and (d), LVEF >40% and LVEF ≤40%

Kaplan–Meier estimates according to electrocardiogram and echocardiography are shown in Figure 3. The results disclosed that low voltage of the limb leads (*log‐rank chi‐square* = 5.624, *p* = .018, Figure 3a), Q‐wave abnormality (*log‐rank chi‐square* = 4.68, *p* = .031, Figure 3b), and LVEDD >60 mm (*log‐rank chi‐square* = 4.434, *p* = .035, Figure 3c) were significantly associated with worse survivals in patients with IIM‐IC. Moreover, Kaplan–Meier survival analysis also revealed that patients with LVEF ≤40% had a significantly higher incidence of all‐cause mortality compared with patients with LVEF >40% (*log‐rank chi‐square* = 5.065, *p* = 0.024, Figure 3d). The survival time was 4.250 (2.925–6.475) years for IIM‐IC with LVEF ≤40%, and 5.00 (2.475–7.875) years for IIM‐IC with LVEF >40%.

## 4 DISCUSSION

Although HF is a common and potentially fatal complication of IIM, studies are limited for patients with autoimmune disease associated IC. Additionally, it is crucial to distinguish these patients from those with primary DCM, as their management may differ. For instance, immunosuppressive therapies may have benefits on improving cardiac function and prognosis through reduction of active myocardial inflammation, beyond that of standard HF medications. Hence, it is crucial for cardiologists to differentiate IC from primary DCM. Here, we described the electrocardiographic and echocardiographic features of patients with IIM‐IC, reporting high frequencies of Q‐wave abnormality, poor R‐wave progression on precordial leads, atrial fibrillation, and ventricular arrhythmia. Specifically, we found that Q‐wave abnormality and reduced LVEF (≤40%) were independent predictors for adverse events and shorter survivals.

The electrocardiogram is an initial, sensitive, and noninvasive examination to identify myocardial involvement in patients with IIM. Electrocardiographic alterations were reported in 30%–80% of cases of IIM (Stern et al., 1984). Rhythm and conduction abnormalities were concluded as the most frequently reported cardiac abnormalities in IIM (Diederichsen, 2017). Especially, left anterior hemiblock and right bundle branch block were considered as the most represented alterations (Mann et al., 2014). On the other hand, although premature ventricular contraction was frequent, ventricular tachycardia had been documented rarely in PM previously (Adler et al., 2002, Lundberg, 2006). However, we found more than two thirds of our patients suffered from ventricular tachycardia, which was a risk factor of sudden death in the general population, suggesting it as a sensitive clinical marker to reflect seriously involved myocardium (Diederichsen, 2017).

We observed that the abnormal Q waves were mainly distributed in leads of anterior wall, inferior wall, and interventricular septum. A British retrospective study once investigated adults with IIM (*N* = 774) matching with the general population (*N* = 7923), discovering an increased risk of myocardial infarction in patients with IIM (*adjusted HR* = *3.89*) after a follow‐up of 15 years (Rai et al., 2016). Abnormal Q waves in adjacent leads usually indicate a prior history of myocardial infarction, and the scarring area might become an origin of ventricular arrhythmia, the latter of which could lead to sudden cardiac death. Considering that all the involved patients had be excluded from coronary artery disease through coronary angiography, the Q‐wave abnormality in our patients could not be explained by “type 1 myocardial infarction.” It is more accurate to describe the Q waves as an abnormal repolarization pattern, suggesting an occurrence of transmural myocardial damage in patients with IIM‐IC. Moreover, the frequency of Q‐wave abnormality was associated with adverse outcome, which was reported the first time in patients with IIM, indicating that the myocardial involvement might lead to cardiogenic re‐admission or cardiogenic death. Based on our analysis, electrocardiogram may help to identify IIM patients with potential myocardial involvement, who may be at higher risk for heart failure hospitalization and/or death.

Similarly, echocardiography might serve as a noninvasive and practical test for further risk stratification after initial evaluation with troponin and electrocardiogram. According to our study, left ventricular diastolic dysfunction was present in 55.8% and reduced LVEF in 50.5% of patients diagnosed with IIM‐IC. Additionally, we demonstrated abnormalities in ventricular size, wall motion, pulmonary artery pressure, and pericardium in patients with IIM‐IC. The possible pathophysiology of ventricular dysfunction includes (i) myocardial inflammation leading to myocyte degeneration and interstitial fibrosis (Schwartz et al., 2016), and (ii) alteration of microcirculation, hyperplasia of intima, and sclerosis of tunica media (Mann et al., 2014). Inflammation can be determined by endomyocardial biopsy meeting the definition of myocarditis, or by CMR imaging (Mavrogeni et al., 2016) demonstrating characteristic myocardial edema and fibrosis (Pipitone, 2016). Considering the availability, safety, and cost, echocardiography may be a more feasible examination for initial assessment of IIM‐IC.

In a prospective research involving 91 subjects with PM and DM, 22 cases died after a median follow‐up of 8.7 years. The heart involvement (*HR* = 1.8) was the independent risk factors of mortality (Danieli et al., 2014). Although cardiac manifestations in IIM have been described as potentially lethal (Dilaveris et al., 2012), the epidemiology of IIM‐IC and prognostic factors of poor outcome have not previously been well established. We therefore explored longitudinal follow‐up of these rare cases, analyzing overall and cardiac‐specific outcomes. Interestingly, we found that the adverse events were seen in more than 50% of our patients after a median follow‐up of 2 years, while the proportion of cardiac death in all‐cause death was as high as 90.9%. Especially in the cases with reduced LVEFs, primary and secondary endpoints were both increased significantly. Additionally, survival time was decreased significantly in IIM with reduced LVEF, dilated left ventricle (LVEDD >60 mm), Q‐wave abnormality, and low voltage of the limb leads. Since cardiac involvement is the leading cause of death in IIM, we suggest enhanced surveillance of patients with IIM‐IC, particularly if Q‐wave abnormality or reduced LVEF are present.

There were several limitations to this study. First, this was a single‐center, retrospective study. Second, medication interventions might influence the overall outcome; however, they were not adjusted in the regression analyses as confounding factors, because of the limited sample size, complicated, and personal‐specialized immunosuppressive therapies, and the fact that there was no management guideline or expert consensus on IIM‐IC.

## 5 CONCLUSION

In our retrospective study of patients with IIM‐IC, we found that Q‐wave abnormality and LVEF ≤40% were associated with adverse clinical events as well as all‐cause mortality. We therefore recommend enhanced attention to diagnosis, management, and surveillance of these patients, including referral to cardiology, close follow‐up, and consideration of immunosuppressive therapy.

## CONFLICT OF INTEREST

Prof Ligang Fang has grant support from the Chinese Academy of Medical Sciences (2019‐I2M‐2‐003), Dr Yingxian Liu has grant support from the National Natural Science Foundation of China (82000470), and the other authors report no conflicts.

## CONSENT FOR PUBLICATION

Not applicable.

## AUTHOR CONTRIBUTIONS

Ligang Fang and Wei Chen performed conception and design; Ligang Fang and Yingxian Liu involved in administrative support; Xue Lin, Yanlin Zhu, and Wei Wu involved in provision of study materials or patients; Xiaohang Liu and Fuwei Jia performed collection and assembly of data; Yingxian Liu and Wei Wu performed data analysis and interpretation; Yingxian Liu drafted the manuscript; Ligang Fang, Jeffrey Hsu, and Wei Chen revised the manuscript. All authors approved the final version of manuscript.

## ETHICS STATEMENT

This study is approved by the Ethics Committee of the Peking Union Medical College Hospital and has been registered in Clinical trials (NCT 03885375).

## Supporting information

Fig S1

Supplementary Material

## Data Availability

The data that support the findings of this study are available from the corresponding authors upon reasonable request.
