# Supplementary figures and images for: Electrophysiological, structural, and functional disorders in patients with inflammatory cardiomyopathy secondary to inflammatory myopathy
Source: Ann Noninvasive Electrocardiol. 2022 Feb 20;27(4):e12938. doi: 10.1111/anec.12938 (PMC9296788; doi:10.1111/anec.12938)

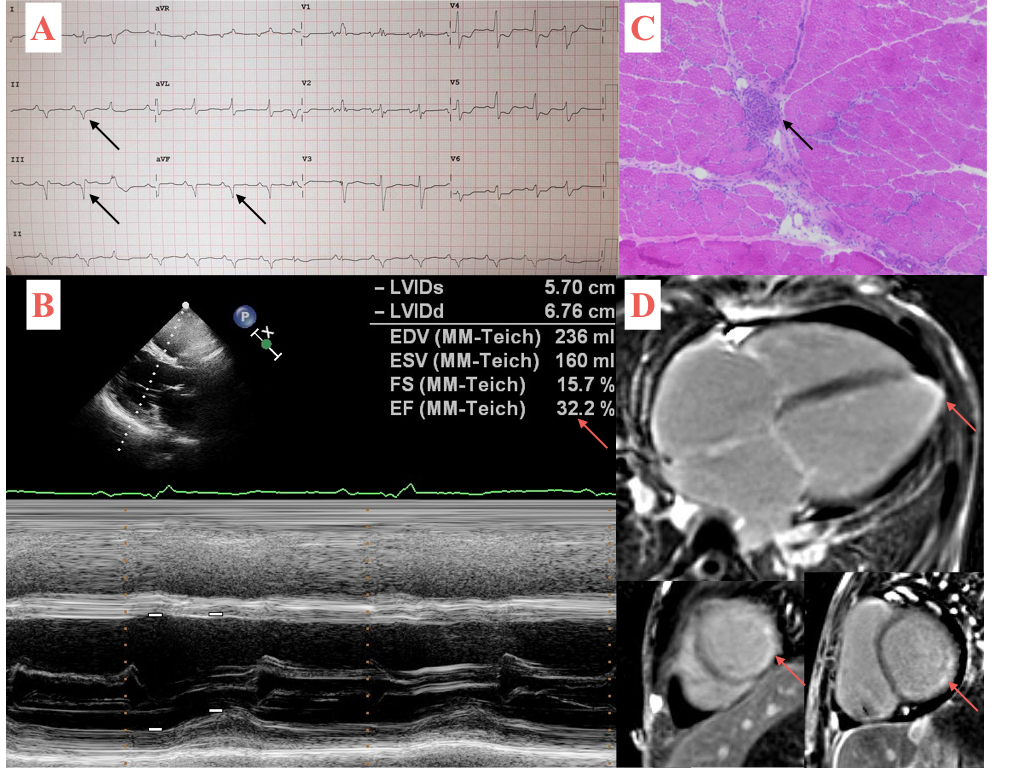

Supplement: Supplementary file 1 — Fig S1 [file ANEC-27-e12938-s001.tiff]
