# Supplementary material for: Electrophysiological, structural, and functional disorders in patients with inflammatory cardiomyopathy secondary to inflammatory myopathy
Source: Ann Noninvasive Electrocardiol. 2022 Feb 20;27(4):e12938. doi: 10.1111/anec.12938 (PMC9296788; doi:10.1111/anec.12938)
Supplement: Supplementary file 2 — Supplementary Material [file ANEC-27-e12938-s002.docx]

**Supplementary Figure 1** Electrocardiogram (A), result of quadriceps muscular biopsy (B), echocardiography (C), and CMR (D) of a 46-year-old male with progressive dyspnea for 2 years. A, electrocardiogram showed left axis deviation, intraventricular and right bundle branch blocks, poor R-wave progression on precordial leads and Q waves in the II, III, and AVF leads (black arrow). B, echocardiography showed left ventricular end-diastolic diameter of 57 mm, and left ventricular ejection fraction of 32% (red arrow) with diffuse wall motion abnormalities in the parasternal left ventricular long axis view. C, quadriceps muscular biopsy showed typical muscle pathology of DM, including perivascular and perimysial inflammatory infiltrations (black arrow), as well as perifascicular necrosis, which indicated dermatomyositis-associated cardiomyopathy. D, CMR revealed four-chamber enlargement, diffuse left ventricular wall motion abnormality with reduced systolic function (LVEF = 27.8%), patched T2-weighted sequences indicating myocardial edema and widespread gadolinium enhancement sequences (red arrow) indicating myocardial fibrosis.
